# Self-Reported Antibiotics Usage, Allergies and Resistance of Albanian Patients from a Dental Perspective: A Preliminary Questionnaire-Based Survey

**DOI:** 10.3390/antibiotics13111057

**Published:** 2024-11-07

**Authors:** Blerina Zeza, Nisrina Kraja, Valbona Disha, Erdita Cenameri, Esat Bardhoshi

**Affiliations:** 1 Department of Periodontology, Faculty of Dentistry, Albanian University, 1001 Tirana, Albania; rinakraja123@gmail.com; 2 Department of Dentistry, Albanian University, 1001 Tirana, Albania; v.disha@albanianuniversity.edu.al; 3 Independent Researcher, 80331 Munich, Germany; erdita.cenameri@gmail.com; 4 Faculty of Dental Medicine, University of Medicine, 1005 Tirana, Albania; ebardhoshi@gmail.com

**Keywords:** antibiotics, antibiotic allergy, antibiotic resistance

## Abstract

Objectives: The paper aims to assess the level of antibiotic use and the antibiotic types used generally in dentistry and identify self-reported allergies and resistance related to them. Methods: The data were collected between March and May 2024 via an electronic questionnaire on self-reported antibiotic usage, antibiotic allergies and resistances among individual in Albania. Results: A total of 477 individuals (83% females, 17% males) with a mean 33 ± 13 (min 17; max 73) years of age completed the questionnaire. Overall, 88% of the population reported having used antibiotics before the questionnaire, among whom 56% used them for dental reasons. An average of 5% reported being allergic, mainly to the penicillin group, while 5% claimed to have undergone an antibiogram analysis before taking the prescribed antibiotics, with most of them showing resistance to the penicillin group. Conclusions: Based on the findings of this study, the high prevalence of antibiotics usage in dentistry encourages further deepening studies and training adapting global guidelines for antibiotics indications in dentistry to the level of antibiotics allergies and resistances of this specific country.

## 1. Introduction

Since penicillin’s introduction in 1928 by Alexander Fleming, antibiotics use in medicine has saved millions of lives and significantly impacted dentistry by improving the management of post-surgical infections and other oral bacterial diseases [1,2,3].

Following World War II, dentists began using antibiotics not only to treat infections, but also as a prophylactic treatment to prevent infections in high-risk patients. In the 1960s, guidelines for prophylactic antibiotic use were developed and continuously revised over time [4,5,6]. The main reason for guideline revision is antimicrobial resistance (AMR), which is one of the major global threats to public health, directly responsible for 1.27 million global deaths in 2019 and contributing to 4.95 million deaths [7].

Approximately 10% of antibiotic prescriptions worldwide are provided by dentists, whether for prophylactic or therapeutic purposes, and are not always considered appropriate, leading to excessive or incorrect antibiotic use in dental practice [8,9]. Efforts to promote appropriate antibiotic use and stewardship in dentistry have increased worldwide [10,11].

The American Dental Association (ADA) guidelines’ most recent revision reported in 2021 indicates prophylactic antibiotic use prescribed from dentists only in patient at risk of endocarditis due to “prosthetic cardiac valves, including trans catheter-implanted prostheses and homografts; prosthetic material used for cardiac valve repair, such as annuloplasty rings and chords; a history of infective endocarditis; a cardiac transplant with valve regurgitation; the congenital (present from birth) heart disease (shunts valvular regurgitation)” only when the dental procedures involve manipulation of gingival tissue or the periapical region of the teeth or perforation of the oral mucosa [12]. Furthermore, the ADA recommends the use of antibiotic prophylaxis prior to the dental procedure or within 2 h from it in case the patients forgot to premedicate. It also excludes clindamycin as an alternative to amoxicillin or ampicillin in individuals with allergies to these drugs, and in case the patient is already taking antibiotics, prophylaxis is advised to be performed using a different antibiotic family [12]. When treating patients in systemic condition or undergoing treatments that compromise their immune response, most studies support preoperative prophylactic antibiotic administration in oral and maxillofacial surgery [12,13].

The therapeutic prescription of systemic antibiotics in oral infections is carried out empirically and is limited to infections associated with systemic signs such as elevated body temperature, lymphadenopathy and lockjaw, acute infection conditions such as necrotizing ulcerative gingivitis, stage III-grade C/incisor-molar pattern periodontitis (formerly referred to as localized aggressive periodontitis), acute periapical abscess, cellulitis, periodontal abscess, pericoronitis, periimplantitis, and infection of deep fascial layers of the head and neck [8,13,14].

Evidence demonstrates that use of antibiotics for therapeutic purposes was unnecessary in 80% of cases in a British study, and for prophylactic purposes, it was similarly inappropriate in 80% of the cases in US [13,15,16].

Săndulescu et al. (2024) listed the most recurrent themes for inappropriate antibiotic prescription reported in the literature as follows: (i) antibiotic prescription to delay or avoid dental treatment; (ii) decision making on guidelines evidence rather than on individual testing, due to the difficulty to establish the exact etiology of most dental infections; (iii) the lack of continuous training and relying on past education; (iv) too many guidelines, leading to confusion and difficulty accessing the guidelines; (v) psychological factors, including pressure to prescribe and the weekend effect (prescribing antibiotics to avoid having complications during the weekend); and (vi) not considering the contribution of their prescriptions to AMR [11].

Studies on antibiotics usage and resistance in Albania are a few and mainly concern antibiotics usage in hospitals, reporting prolonged post-operative antibiotic usage in prevention of surgical-site infections [17]. The Institute for Health Metrics and Evaluation reported in 2019 that Albania recorded 339 deaths directly attributable to antimicrobial resistance (AMR) and 1300 deaths associated with AMR [18]. Recently, it has been reported that among the top 10 antibiotics used in Albania, the number of “Watch” antibiotics from 2011 to 2021 has increased from 10% to 82% of the list [19]. “Watch” antibiotics were defined by the WHO in 2022 as “antibiotics that generally have a higher potential for the selection of AMR and are commonly used in sicker patients hospitalized. Their use should be monitored carefully in order to avoid overuse” [20]. One of the most used antibiotics from 2011 to 2021 in Albania was Amoxicillin, or its combination with clavulanic acid, both “Access” antibiotics [19]; the same is widely prescribed by Albanian dentists [21]. In case of allergy or resistance to penicillin, Azithromycin was reported to be Albanian dentists’ second choice [21], which is a “watch antibiotic” and has been noticed to have an increased use since 2019. In 2020 and 2021, it was the most used antibiotic in Albania [19].

Evidence on the relevance that dentistry has in antibiotic prescription in Albania is relatively limited. In 2022, a six-month study conducted among dentists working in private practices and university dental clinics reported that the number of prescriptions given by Albanian dentists to adult patients was high, counting for a mean of eight prescriptions per week [21].

On the other hand, dental patients may not correctly adhere to prescribed antimicrobial treatment, exacerbating the overuse and/or misuse of antibiotics in dentistry [22]. To the authors’ knowledge, evidence is not available on the information patients received nor the kind of information they report to dentists when asked during their anamnesis.

Thus, the main aim of this preliminary survey was to observe from a patient perspective what role dentists and dental or oral diseases they suffered played during the usage of the prescribed antibiotics and the exchange of information between the two parties prior to antibiotic prescription.

## 2. Results

### 2.1. Population Description

A total of 477 individuals (83% females, 17% males), with a mean age of 33 ± 13 (min 17; max 73) years, completed the questionnaire. 

Most of the population (88%) had used at least once antibiotic. Overall, 56% of the population who had used antibiotics reported having used them for dental problems, among which the most common was dental pulp infections (76%). Other dental reasons are reported in Table 1.

Patients were asked separate questions about antibiotics administration after a professional tooth cleaning or because of “bleeding gums” reported to the dentist, and the answers were positive for 7% and 12% of the cases, respectively. In this regard, only 27% of the entire population was ever informed by a dentist about periodontal diseases. 

**Figure 1:**
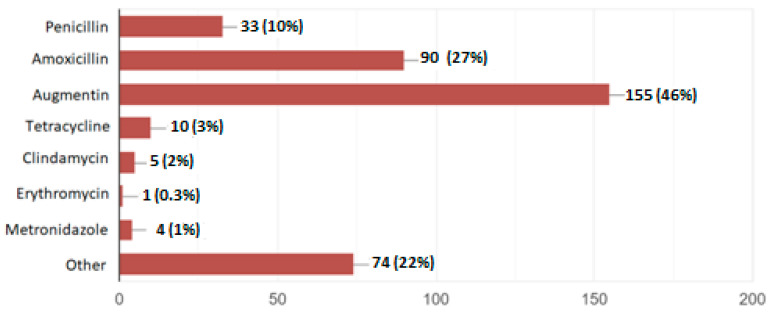
The type of antibiotics prescribed by the dentists in the study among those who reported having ever used antibiotics.

The most commonly prescribed antibiotic was reported to be Amoxicillin/clavulanic acid (46%) (Figure 1). 

### 2.2. Antibiotic Resistance

Among participants that reported having used antibiotics, only 79% had heard of antibiotic resistance and 31% had been informed about it by their dentists. When asked if they had ever experienced that the antibiotics taken did not improve the dental or oral symptoms or signs the antibiotics had been prescribed for, 7% reported having experienced this. The questionnaire also included a question regarding undergoing an antibiogram before prescription of a specific antibiotic, to which only 21 (5%) out of 421 respondents who have used antibiotics responded that an antibiogram was used to determine the appropriate antibiotic. Among those, 6 out of 21 (29%) reported being resistant to the penicillin group. 

### 2.3. Antibiotic Allergy

Among the population completing the questionnaire and reported ever taking antibiotics during their life, an average of 5% reported being allergic, mainly to the *Penicillin* group. When asked if their dentist had ever asked about being allergic or not to any antibiotic, 70% reported being asked. 

## 3. Discussion

This study was principally conducted to observe antibiotics usage for dental-related problems, antibiotics-related allergies, and antibiotic resistance in a self-report form in order to determine the role of dentists in informing and prescribing antibiotics to patients in the present population in Albania.

A large portion of the participants (88%) had used antibiotics at least once during their life as far as they could remember. Among those who have used antibiotics, 56% have used them at least once for dental-related problems, mostly dental pulp infections. Worldwide, dental practices account for a non-negligible percentage of total antibiotic usage in human medicine, with an increasing tendency for prescriptions after the COVID-19 pandemic [11]. Despite the high incidence of odontogenic infections and the high percentage of cases of dental pain due to pulp infections, which require operative intervention by dentists rather than an antibiotic treatment, there are no specific criteria for prescribing antibiotics in this area [8]. The target population in the survey does not permit an evaluation of the appropriateness and correctness of the prescriptions, as responses were self-reported. However, given the aforementioned evidence, inappropriate prescription may be indirectly assumed. Shpati et al. in 2022 reported that an average of 37.01% of dentists in Albania had insufficient knowledge on antibiotic prescription and a self-expressed need for trainings in this regard [21]. Incorrect and inappropriate antibiotic prescription by dentists have been reported in other countries as well as in the UK (81% of therapeutic antibiotic prescriptions) and the USA (81% of prophylactic antibiotic prescriptions) [11]. The recommended duration of antibiotics therapy in dentistry is approximately 3–7 days, with a re-evaluation within 3 days (for example, in-person visit or phone call). Dentists should instruct patients to discontinue antibiotics 24 h after their symptoms resolve, irrespective of re-evaluation after the third day [12,14]. 

A small percentage of the population reported using antibiotics after a professional plaque and calculus cleaning or for bleeding gums. Antibiotics indication for periodontal diseases and conditions are very restricted [13,14]. Periodontal infections have specific characteristics: (i) The main etiologic factor known is dental plaque, a biofilm that represents a protective surrounding for periodontal pathogens even from antibiotics used locally, or systemically, favoring resistance toward antibiotics used. The mechanical disruption of the biofilm is essential prior to antibiotics use in case where they are indicated. (ii) Dental plaque is localized in the dental surface, which cannot desquamate to reduce its accumulation. Furthermore, the teeth present at the crown level present different retention sites, favoring the accumulation of dental plaque despite its removal. This emphasizes the importance of an everyday oral hygiene routine. Finally, (iii) dental plaque is localized outside the organism, and thus the immune system cannot exert its maximum protection potential [23]. A systemic antibiotic prescription is recommended only for necrotizing gingivitis and periodontitis, a stage III-grade C and incisor–molar periodontitis (previously referred to as aggressive localized periodontitis), periimplantitis, and pericoronitis [13].

The most prevalently used antibiotic prescribed by dentists in the present study was amoxicillin/amoxicillin with clavulanic acid (Augmentin), and the reported resistances were predominantly from the penicillin group, even though conclusions are difficult to draw with the small number of resistances reported. Similar results on the prevalent prescription of the aforementioned antibiotics have been reported among dentists in Albania [21] and among doctors in Albanian hospitals [19] and worldwide [13]. This result is supported by the evidence that in most dental indications, the determination of antibiotic prescriptions was conducted in an empiric way [11]. 

Ideally, antimicrobial susceptibility testing such as antibiograms should be performed for patients in potential need of an antibiotic prescription. Antibiograms in dentistry have their own cost-related limitations, and it is difficult to obtain a sample when infections and the cases of antibiotic prescription are dental emergencies. For the above reasons (among others), in dentistry, antibiotic prescriptions are mostly conducted in an empirical way [11]. According to authors, the usage of an antibiogram should be useful to more accurately prescribe antibiotics, especially in the case of periodontal infections or hospitalized patients, still remaining in the indication list of systemic use of antibiotics in dentistry and where samples for examination can properly obtained. Among the participants of the study population reporting the usage of antibiotics, only 5% were submitted to an antibiogram, with 29% reporting being resistant to penicillin. 

The 5% of respondents who self-reported an allergy to penicillin in the study population are, however, a smaller percentage compared to the results reported in the United States, with around 10% of the population (32 million in total) reporting an allergy [24]. Patients labeled as penicillin-allergic could be falsely identified as such, as an intolerance to penicillin sometimes shows symptoms mistakenly attributed to an allergic reaction, or their allergy reaction symptoms may have diminished over time; these patients are more likely to be prescribed a broad-spectrum antibiotic, which contributes to antibiotic resistance, affecting both the individual and society [24,25,26]. Overall, 90% of reported allergies are found later to be not allergies and are de-labeled as antibiotics allergies, relying mainly on in vivo testing, including drug provocation and/or skin test; re-testing is considered to be an important pathway against antibiotics resistance [27]. Many individuals, particularly young people with few medical issues or those without any medical problems, do not frequently visit medical clinicians but may see a dentist, placing dental offices at a crucial position in penicillin allergy evaluation [24], including de-labeling at least at an informative level and accompanying the reported process of fear of taking antibiotics even after de-labeling [28]. 

The main limits of the present study are the small targeted population group, lack of representativeness, doubtful reliability of self-reported antibiotic use [29,30], the lack of interaction with the individuals that completed the questionnaire in order to further investigate the responses given, and the lack of verification of the results of a possible antibiogram when reported. The first participants receiving the questionnaire in the survey were dental students whose family and relatives could be assumed have a dental professional background. This could be another limit of the study, underestimating the use of antibiotics as professional dental education tends to have positive effects on dental students’ oral health attitude and behavior and dentists have better oral health than the general population [31,32].

It was noticed from the preliminary survey of this specific population that dentists have an important quantitative (56% use of antibiotics for dental reasons) imprint on antibiotics use, giving them a greater responsibility for their informative role they play with patients and emphasizing the need to update their knowledge through training on the matter. A previous study of Albanian population reported the need for dentist trainings on antibiotics prescription from a pharmaceutical prospective [21]. Given the high prevalence of self-reported antibiotics used for dental pulp infection, authors suggest that these trainings and the information communicated to the patient should include information on the importance of the internal environment along with the microbial etiology of dental diseases. As Bernard and Bechamp postulated, a disease only occurs when the terrain or internal environment of the body becomes favorable to germs, and the germs are subject to modification once the internal terrain changes, so they are more a consequence rather than a cause [33]. Thus, the patient is given the possibility to become aware of the responsibility they have in the healing process and to not rely totally on drugs or pressure their dentist to prescribe antibiotics when they are not needed [11]. 

Furthermore, more representative surveys on antibiotics allergies and resistance in this specific region would be useful to public health policies for antibiotic stewardship. 

## 4. Materials and Methods

This is a questionnaire-based observation of antibiotics used from patients for dental-related problems, which consisted of closed and open-ended questions. The study was conducted following “the Declaration of Helsinki” 1975, revised in 2013, and approved by the Institutional Review Board (Ethic Committee) of Albanian University (protocol code 71, date of approval 14 February 2024). 

The questionnaire was designed using Google Forms program (https://docs.google.com/forms/d/e/1FAIpQLSc980eAymveTZOOQOkvHul6uWO05ciNAH4rckLmbhbdc4IW2g/viewform?fbzx=-2895901124852422916, accessed on 15 January 2024) with the inclusion of (i) introductory instructions, where the purpose of conducting the study and a statement on maintaining the anonymity and confidentiality of the surveyors’ data were provided; (ii) demographic (personal) data; and (iii) questions related to the study topic, which in the present case was the use of antibiotics for dental-related problems, antibiotics resistance, and allergies. It was distributed between March and May 2024 as a link generated by the aforementioned program using e-mails and mobile phones. The operators responsible for the investigations used the contact information they have of the students of Dentistry at Albanian University, Albania, as the first subjects to be contacted and encouraged the distribution of the survey among friends and family for a domino effect. Given the aim of the survey was to evaluate preliminary self-reported use of the antibiotics during one’s life based on what and how far back they remember, particularly in relation to dentists and dental-problems, the population started from the people surrounding close to operators (authors responsible for the investigation and the link distribution) and was followed by encouraging each subject contacted first for recommendations of friends and relatives. No specific inclusion and exclusion criteria were followed.

The questions were formulated based on the literature [8,13,14,15] and authors’ clinical experience, and adapted such that each one could be easily understood despite the level of knowledge and social status of the participant. Each question underwent prior internal testing within a relatively small group of people, where feedback, ideas, and impressions related to clarity and relevance of each question were collected, followed by modifications made to improve the questionnaire. 

The introductory paragraph served as an informed consent statement, with a brief description of the aim of the study and the privacy-related concerns, including usage of the data gathered during the survey, and emphasizing the free-will decision of the participants to continue or not the completion of questionnaire without any consequences. Participants were informed that demographic data would be used only in relation to the purpose of the study. Questions related to the topic of the study were mainly closed questions, giving patients the possibility to more easily provide an answer to the questions, with the assumption that participants did not have any proper dental or medical knowledge. The main question regarded the use of antibiotics, as far as they could remember and identify, and if their use was related to dental problems. The participants were asked to choose from different alternatives offered, the reason they had been prescribed antibiotics, the type of antibiotic prescribed, identify its effectiveness, if they have been asked questions about any allergies or resistance to antibiotics, and if an antibiogram analysis was ever conducted in their case. A couple of questions were dedicated to periodontal diseases. The participants were asked if they were informed on periodontal disease, if they were ever prescribed antibiotics after professional plaque and calculus removal sessions, and if they were ever prescribed antibiotics by their dentists due to bleeding gums. A summary of the questionnaire is available in the Supplementary Materials (Figure S1). 

The statistical analysis performed, in accordance to and conditioned by the nature of the data collected, was a descriptive analysis of the information extracted in an Excel file from the Google Form program in order to analyze the proportion of participants who had used antibiotics, were allergic to them, reported resistances, or who underwent an antibiogram analysis, as well as what was the most prevalently prescribed antibiotic from dentists and the most prevalently reported dental-related reason for antibiotics prescription. 

## 5. Conclusions

Within the limits of this preliminary study, the authors concluded that a relevant percentage of the population has used antibiotics for dental-related problems, mainly in dental pulp infection treatment. The information communicated to patients by dentists needs improvement regarding allergies and resistance. Specific training focused on the importance of the internal environment would increase the awareness of the present population on antibiotic use and on the patient’s responsibility during the healing process. 

## Figures and Tables

**Table 1:** Dental and oral conditions for which the individuals reported having been prescribed antibiotics. The percentages were calculated as the number of subjects reporting antibiotics used for a specific condition over the portion of the population who reported having ever used antibiotics.

Dental and Oral Conditions	Size in Number	Percentage
Dental pulp infection	179	76
Tooth extraction	18	8
Irritation of the mucosa around third molars	13	6
After a surgery	7	3
Abscesses at gingival level	2	1
Face swelling due to dental infection	1	0.4
Granuloma	1	0.4
Pain related to treatment	1	0.4
Other	6	3

## Data Availability

Data are unavailable due to privacy and ethical restrictions.

## Supplementary Materials

The following supporting information can be downloaded at: https://www.mdpi.com/article/10.3390/antibiotics13111057/s1, Figure S1: Summary of the questionnaire distributed to participants.
