# Supplementary material for: Self-Reported Antibiotics Usage, Allergies and Resistance of Albanian Patients from a Dental Perspective: A Preliminary Questionnaire-Based Survey
Source: Antibiotics (Basel). 2024 Nov 7;13(11):1057. doi: 10.3390/antibiotics13111057 (PMC11591203; doi:10.3390/antibiotics13111057)
Supplement: Supplementary file 1 [file antibiotics-13-01057-s001.zip › antibiotics-3248855-supplementary.pdf]

QUESTIONNAIRE ON ANTIBIOTIC USAGE, RESISTANCE AND ALLERGIES

This is a questionnaire that aims at asking for your experience on the use of antibiotics for dental problems. We would be grateful if you answer responsibly. Your answers are anonymous and they will be used for research purpose only respecting your privacy. We thank you in advance for the collaboration!

Nisrina Kraja

Dental student, Albanian University

1. Age:____________
2. Gender: M\ F
3. Level of education: Elementary\ Midschool\University
4. Profession:_______________________
5. Have you ever used antibiotics: Yes\No
6. Have you ever been informed by the dentist that you have periodontal disease or that you could lose your teeth due to periodontal infections: Yes\No
7. Have you ever used antibiotics after professional tooth cleaning: Yes\No
8. Have you ever been prescribed by the dentist due to bleeding gums: Yes\No
9. Have you ever used antibiotics for dental problems: Yes\No
10. If yes, what dental problem: dental pulp infection\tooth extraction\irritation of the mucosa around the third molar\after surgery\abscess at gingival level\swelling of the face due to dental infection\granuloma\pain after treatment\other
11. Which one of the following antibiotics have the dentist prescribed to you: penicillin\amoxicillin\ augmentin\ tetracycline\ clindamycin\ erythromycin\ metronidazole\ other specify
12. Are you allergic to any antibiotic: Yes\No
13. If yes, specify which one:______________________________
14. Have the dentist asked you for allergies to any antibiotic before prescribing it: Yes\No
15. Have you ever heard of antibiotic resistance (when you use it but it might not be effective): Yes\No
16. Have the dentist discuss with you the antibiotic resistance: Yes\No
17. Have you any dental infection that have not responded to the antibiotics used: Yes\No\I do not remember
18. Have you ever done an antibiogram (analysis used to see if the microorganism are resistant or not to any antibiotic): Yes\No
19. If yes, which antibiotic have been test for:_____________________________
20. If you remember, which was the result: Resistant\Not resistant
21. How did the dentist react or what action was taken when the antibiogram resulted resistant:________________________________

Figure S1. Summary of the questionnaire distributed to participants.
